# Vitamin C Supplementation in the Treatment of Autoimmune and Onco-Hematological Diseases: From Prophylaxis to Adjuvant Therapy

**DOI:** 10.3390/ijms25137284

**Published:** 2024-07-02

**Authors:** Stefania Isola, Luca Gammeri, Fabiana Furci, Sebastiano Gangemi, Giovanni Pioggia, Alessandro Allegra

**Affiliations:** 1 School and Operative Unit of Allergy and Clinical Immunology, Policlinico “G. Martino”, Department of Clinical and Experimental Medicine, University of Messina, 98125 Messina, Italy; stefania.isola@unime.it (S.I.); gangemis@unime.it (S.G.); 2 Provincial Healthcare Unit, Section of Allergy, 89900 Vibo Valentia, Italy; fabianafurci@gmail.com; 3 Institute for Biomedical Research and Innovation (IRIB), National Research Council of Italy (CNR), 98125 Messina, Italy; giovanni.pioggia@cnr.it; 4 Division of Hematology, Department of Human Pathology in Adulthood and Childhood “Gaetano Barresi”, University of Messina, 98100 Messina, Italy; alessandro.allegra@unime.it

**Keywords:** vitamin C, autoimmunity, cancer, leukemia, multiple myeloma, lymphoma, dietary integration, micronutrient

## Abstract

Vitamin C is a water-soluble vitamin introduced through the diet with anti-inflammatory, immunoregulatory, and antioxidant activities. Today, this vitamin is integrated into the treatment of many inflammatory pathologies. However, there is increasing evidence of possible use in treating autoimmune and neoplastic diseases. We reviewed the literature to delve deeper into the rationale for using vitamin C in treating this type of pathology. There is much evidence in the literature regarding the beneficial effects of vitamin C supplementation for treating autoimmune diseases such as Systemic Lupus Erythematosus (SLE) and Rheumatoid Arthritis (RA) and neoplasms, particularly hematological neoplastic diseases. Vitamin C integration regulates the cytokines microenvironment, modulates immune response to autoantigens and cancer cells, and regulates oxidative stress. Moreover, integration therapy has an enhanced effect on chemotherapies, ionizing radiation, and target therapy used in treating hematological neoplasm. In the future, integrative therapy will have an increasingly important role in preventing pathologies and as an adjuvant to standard treatments.

## 1. Introduction

Many recent studies have reported the importance of vitamin C (ascorbic acid) for general health and its role in the pathogenesis of various diseases, considering its antioxidant activity and its effects on DNA demethylation. Vitamin C is a water-soluble vitamin obtained through the dietary intake of fruit and vegetables, initially identified as a factor in preventing scurvy disease [1]. Recently, it was recognized for its anti-inflammatory properties. Vitamin C improves immune functions by setting innate and adaptive immune responses, reduces cellular oxidative stress, and acts as a cofactor for several enzymatic reactions. Its pleiotropic use has been proven in treating sepsis and infectious diseases, autoimmune diseases, bone diseases, skin diseases, and cancer [2].

In patients with autoimmune diseases, like Systemic Lupus Erythematosus (SLE), Rheumatoid Arthritis (RA), Systemic Sclerosis (SS), Type 1 Diabetes, and Psoriasis, vitamin C can help reduce inflammation and oxidative stress by reducing the production of pro-inflammatory cytokines and free radicals. Vitamin C and other micronutrients are also involved in regulating epigenetic processes. The effects of vitamin C on immune function are expressed in neutrophils and macrophages. Vitamin C works as an electron donor and antioxidant agent. Moreover, it enhances cell motility, chemotaxis, phagocytosis, reactive oxygen species (ROS) generation, microbial killing, facilitates apoptosis and clearance, and modulates cytokine production [3]. Furthermore, vitamin C acts on lymphocytes T, preserving the immunosuppressive capacity of T-reg [4] by enhancing differentiation, proliferation, and antibody levels [5]. This vitamin is also essential to ensure physiological natural killer (NK) cell development and function and acts on basophils, eosinophils, and mast cells by modulating cytokine production and reducing histamine levels [6].

Concerning cancer, knowledge of vitamin C’s physiological and pharmacological mechanisms has opened new perspectives on its possible use in managing various tumors through its capacity to act on cancer growth and development, and considering further aspects such as its low toxicity and low financial cost [7]. Indeed, vitamin C at a high dosage can modulate the chemotaxis in the tumor microenvironment of immune system cells and enhance the cytotoxic role of CD8 T cells, jointly with immune checkpoint therapy (ICT) in many cancers [8].

Therefore, this paper reports an analysis of vitamin C’s role in cancer, particularly in hematologic tumors, with a short focus on its role in autoimmune diseases like SLE and RA. This work aims to evaluate the possible applications of vitamin C integration to obtain a precision medicine approach that may be a turning point in therapy.

## 2. Vitamin C and Autoimmune Diseases

Several studies have documented the essential roles of oxidative stress and free radicals in many human diseases [9], such as Alzheimer’s disease, Parkinson’s disease, amyotrophic lateral sclerosis [10], cardiovascular disease [11], allergies [12], diabetes [13], cancer [14] and autoimmune diseases [15].

The state of oxidative stress leads to an abnormal accumulation of free radicals, which harm the body’s cells and tissues [16].

Among autoimmune diseases, we focused our attention on SLE and RA.

SLE is the autoimmune disease prototype, with incidence of 0.3–31.5 in 100,000 per year [17]. The pathogenesis of the disease results from complex interactions between a genetic substrate and epigenetic modifications, as well as hormonal and environmental factors [18]. It is characterized by a loss of tolerance to self-antigens, leading to an abnormal response by B and T cells, lymphoproliferation, and overproduction of anti-nuclear autoantibodies, proinflammatory cytokines, and immune complexes. These events start and amplify inflammation, contributing to SLE’s clinical manifestations and affecting various tissues and systems (skin, joints, heart, lungs, kidneys, circulating blood cells, and brain) [19].

Oxidative stress plays a central role in exacerbating inflammation and disease activity.

Oxidative alterations of proteins, lipids, and DNA can contribute to a dysregulation of the immune system, leading to the activation of molecular mechanisms such as NETosis, the mTOR pathway, and the unbalanced differentiation of T cells [20]. All these mechanisms contribute to the worsening of the inflammatory state.

There is much evidence regarding vitamin C’s potential therapeutic role in SLE. Vitamin C has a scavenger action on ROS, which is responsible for the uncontrolled lipid peroxidation of blood cells and other blood components [21].

In addition to the role of ROS, neutrophils are another critical element in the pathogenesis of damage in SLE. These cells can produce extracellular traps (NETs), releasing DNA strands associated with histones and enzymes like proteases and oxidases. Under physiological conditions, NETs trap entangling pathogens; however, they can also be released in other conditions, promoting inflammation and damaging host tissues [22].

NETs are a significant source of autoantigens and are involved in the pathophysiology of various autoimmune diseases, such as SLE, RA, vasculitis, psoriasis, and antiphospholipid syndrome [23]. Studies on in vitro models have demonstrated the importance of vitamin C in regulating the NET formation and the NETosis process [24]. The same authors demonstrated the inhibitor activity of vitamin C on NETosis in polymorphonuclear neutrophils from healthy donors [24].

Based on the well-established connection between nutrition and immunological function, recent studies have investigated the impact of diet on triggering or altering the course of the disease.

Constantin et al. [25] emphasize the beneficial role of personalized diet in patients with SLE. A personalized diet can help maintain homeostasis, improve disease remission, and limit the adverse effects of medications, enhancing the physical and mental well-being of these patients.

In particular, vitamin C supplementation, in addition to preventing oxidative stress, reduces inflammation and determines a reduction in the levels of autoantibodies such as anti-dsDNA. According to the authors, vitamin C should be integrated into the diet of SLE patients (maximum dose of 1 g/day) individually or in combination with vitamin E [25].

Vitamin C intake is inversely associated with the risk of disease reactivation, decreasing oxidative stress, and repressing autoantibody production [26]. This evidence supports the role of vitamin C intake in preventing SLE flares, and it was reconfirmed in 2003 with a four-year prospective Japanese study on 216 patients [27].

SLE is also associated with an increased risk of severe cardiovascular disease. In addition to the classic risk factors, the risk is also increased in these patients due to the excessive production of free radicals, altered lipid metabolism, and chronic inflammation [28].

Tam et al. [29] conducted a double-blind, randomized, placebo-controlled study on 39 SLE patients receiving vitamin C (500 mg of vitamin C and 800 IU of vitamin E). Levels of malondialdehyde (MLD), allantoin, erythrocyte superoxide dismutase (SOD), glutathione peroxidase, and serum concentrations of vitamin C and vitamin E were measured. After 12 weeks of vitamin integration, it was observed how the combination of vitamin C and vitamin E administration decreased lipid peroxidation. However, vitamin supplementation does not affect endothelial function in SLE patients [29].

Epigenetics is another key element in the pathogenesis of SLE and other autoimmune diseases. These mechanisms can influence gene expression or repress the target cells and tissues involved in the disease, working as effectors in regulating both adaptive and innate immunity. DNA methylation, histone modifications, and noncoding RNA profiling are central epigenetic mechanisms.

It appears that diet is an important factor since it is directly linked to epigenetic alterations and the effects of bioactive compounds on the epigenome. Therefore, an epigenetic diet could offer an approach to preventing, treating, and diagnosing future diseases. The cross-link between epigenetic mechanisms on the predisposition and development of SLE and the influence of dietary factors on regulating epigenetic modifications could positively impact SLE patients [30].

SLE patients present decreased levels of vitamins, methionine, and other methyl donors compared with healthy individuals. Vitamin C seems to enhance the activity of Ten eleven translocation enzymes (TETs), a family of enzymes implicated in DNA demethylation mechanisms [31]. Specifically, these enzymes are involved in the expression of the *FOXP3* gene.

Foxp3 is a transcription factor involved in regulatory T cell (T-reg) development and regulation. These cells are fundamental in regulating autoimmune processes and the homeostasis of the immune system. Therefore, implementing vitamin C could stabilize the expression of *FOXP3* and promote the activity and efficiency of T-reg cells.

The treatment of these patients focuses primarily on immunosuppressive and biological agents that inhibit autoreactive B cells, stop cytokine signaling, and favor the development of T-reg cells.

The literature supports diet therapy and vitamin C supplementation as promising approaches to inflammation in patients with SLE and may offer these patients a better quality of life [32]. A low-calorie and low-protein diet, with high contents of fiber, polyunsaturated fatty acids, polyphenols, and vitamins, guarantees an appropriate intake of macronutrients and micronutrients able to positively modulate disease activity by acting on the inflammatory microenvironment and immune cells [33,34].

RA is a multifactorial autoimmune disease characterized by chronic inflammation with joint and, in some cases, extra-articular involvement. Inflammation causes synoviocyte hyperplasia, destroying bones and joints [35].

The global incidence rate of RA is about 0.5% to 1%, and the prevalence in females is three times higher than in males [36].

The possible extra-articular involvement can lead to multiple comorbidities, which include cardiovascular diseases, interstitial lung diseases, osteoporosis, infections, fatigue, and depression, with an increased mortality rate [37].

The disease is also associated with an increased incidence of lymphomas, leukemia, and lung cancer [38].

Multiple genetic and environmental factors have been associated with an increased risk of RA. A high association was observed with female sex, a family history of RA, exposure to tobacco smoke, and dietary habits [39].

The pathogenesis of the disease is complex. The inflammatory process begins at the synovial level, where macrophages produce pro-inflammatory cytokines. T cells, neutrophils, and plasma cells are also involved, perpetuating inflammation and producing rheumatoid factor (RF) and anti-citrullinated protein antibodies [40].

In the pathogenesis of RA, considerable weight is given to the production of free radicals in the inflammatory microenvironment and to the decrease in antioxidant levels, which may be one cause of the worsening of the disease symptoms and could play an essential role in RA development [41]. Antioxidant supplements with vitamin C have been suggested to improve symptoms by reducing disease-related oxidative stress. Karatas et al. [42] wanted to study the state of RA patients’ oxidative and antioxidant systems, identifying the differences compared to healthy subjects. The study included 22 RA patients and 20 healthy subjects, and the levels of MLD and antioxidant vitamins (A, E, C) were measured. Furthermore, the activity levels of antioxidant enzymes in the erythrocytes were estimated.

Analyses showed that MLD levels in RA patients were significantly higher than in healthy subjects. At the same time, vitamins A, E, and C and antioxidant enzyme activities were lower in RA subjects [42].

Also, epidemiologic investigations have shown a reverse association between dietary intake of antioxidants and disease incidence. The EPIC–Norfolk study evaluated 73 patients with inflammatory polyarthritis (IP) who consumed smaller amounts of fruit and vitamin C than controls. The lower intakes of fruit, vegetables, and vitamin C were associated with an increased risk of developing IP [43]. On the other hand, the large Danish prospective study concluded that dietary factors are irrelevant as risk factors for RA, although they might be important before clinical diagnosis [44]. A cross-sectional study investigated the association between the intake of micronutrients with antioxidant effects and inflammatory and antioxidant markers in patients with active RA. The authors concluded that some antioxidant micronutrients have an essential role in ameliorating inflammatory conditions and enhancing the antioxidant enzymes’ activity in patients with RA [45].

The current management strategy for RA focuses on reducing joint symptoms and slowing its progression to disability. Apart from using pharmaceutical drugs such as non-steroidal drugs, glucocorticoids, and biological agents, identifying common dietary substances that can offer protection or modulate the onset and severity of the disease may have significant health implications. Dietary intake or integration with antioxidants may be helpful as a complementary therapy in controlling oxidative stress in patients with RA and obtaining better clinical outcomes [46].

In the past 40 years, different trials have been carried out to evaluate the effect of dietary antioxidants and antioxidant-rich diets in controlling clinical outcomes of patients with RA. In an open label randomized comparative study, 60 patients were randomly divided in the study or control group. The control group was treated with Hydroxychloroquine 400 mg and Indomethacin 25 mg. The study group received the same treatment in association with Vitamin C 500 mg and Vitamin E 400 mg. After eight weeks of therapy in the study group, a statistically significant clinical improvement and a reduction in inflammatory markers were observed [47]. Nourmohammadi et al. [48] showed that supplementation with 300 mg of vitamin C, 5 mg of zinc, and 25,000 International Units of vitamin A for 12 weeks reduced RA activity.

**Figure 1:**
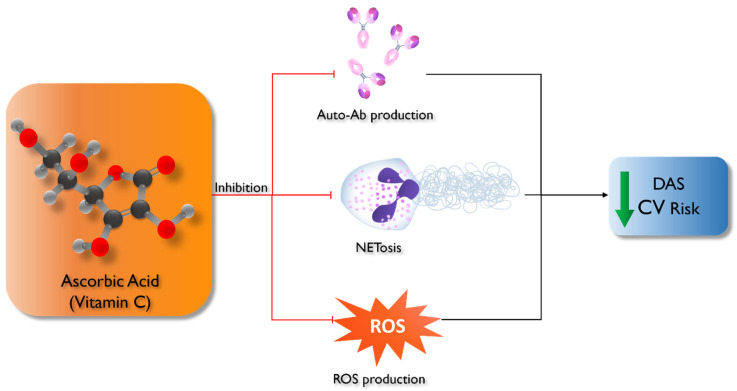
Effects of vitamin C supplement in patients with autoimmune diseases (SLE and RA). Vitamin C influences the immune system by inhibiting plasmacells’ production of self-reactive antibodies. Furthermore, vitamin C has an inhibitory effect on NETosis. The effects on SOD are well known, with consequent reduction in oxidative stress. The final effects are a reduction in DAS (disease activity score) and cardiovascular risk.

The main effects of vitamin C in the genesis of autoimmune diseases are summarized in Figure 1.

## 3. Vitamin C and Hematological Neoplastic Diseases

In recent years, the study of vitamin C’s physiological and pharmacological characteristics and mechanisms has highlighted its possible use in managing various tumors, given its action on various cancerous cell processes, such as cancer growth and development.

Many studies reported that the assumption of vitamin C at a high dosage is related to a decreased risk of developing cancer of the oral cavity, stomach, esophagus, pancreas, cervix, breast, and rectum [49,50,51].

These results have been explained over time with various hypotheses aimed at supporting the positive and protective role of vitamin C in patients with cancer. The protective effects of vitamin C have been traced back to its immunomodulatory action, its ability to promote collagen synthesis, and its inhibitory effects on enzymes involved in the mechanisms of metastasis or on potentially oncogenic viruses. Furthermore, vitamin C can increase sensitivity to chemotherapy and reduce its toxicity [52].

Vitamin C also contributes to iron metabolism, the values of which are altered in the cells of some tumors (e.g., breast cancer, prostate cancer, and lymphoma) due to increased iron absorption processes and reduced iron disposal [53].

In particular, high-dose vitamin C may elevate cellular labile iron pool (LIP) concentrations, causing cell death through ferroptosis, an iron-dependent type of controlled cell death [54]. Moreover, vitamin C produces H_2_O_2_ in the extracellular space, consequently inhibiting tumor cells [52,55,56].

Bowie et al. [57] reported the role of vitamin C in the suppression of the nuclear factor kappa-light-chain-enhancer of activated B cell (NF-κB) signaling, which regulates various genes involved in inflammation and immunological pathways, as well as in cell proliferation, differentiation, and survival. Finally, the role of high-dose vitamin C in promoting the production of 5-hydroxymethylcytosine (5hmC) and DNA hypomethylation could be fundamental for preserving genomic integrity and preventing and treating cancer [58].

All these mechanisms are crucial to understanding vitamin C’s relevance in neoplastic diseases, particularly hematological neoplastic diseases, which are the subjects of this section.

In recent years, vitamin C’s importance has been investigated in relation to hematological neoplastic disorders. It is essential for the immune system and blood-forming cells to mature. Vitamin C can also influence phagocytic cells such as neutrophils, and it can enhance chemotaxis, phagocytosis, the generation of ROS, and, eventually, the removal of microorganisms [6]. It is also necessary for apoptosis and macrophages to remove exhausted neutrophils from infection sites [3,6,23]. Numerous investigations have revealed a correlation between vitamin C administration and the growth of granulocytes [59,60]. Moreover, vitamin C stimulates the proliferation of NK and T cells [61,62]. Additionally, reduced vitamin C levels within cells may promote the transformation of healthy cells into leukemia cells [63] and vitamin C administration could improve leukemia patients’ health-related quality of life [64]. Preliminary data indicate that vitamin C inhibits cellular signaling and enhances autophagy, which might decrease leukemic cell survival and increase sensitivity to conventional chemotherapy [65,66,67]. In the following sections, we will assess variances in vitamin concentrations relative to the normal population, investigate the effects of vitamin C in various hematological pathologies, explore its potential roles in the development and progression of the disease, and examine the effects of supplementation in the pathologies.

### 3.1. Vitamin C and Acute Leukemia

It has been suggested that diet may lower the incidence of adult leukemia [68]. In vivo tests on pregnant rats fed N-nitroso precursors, namely nitrites and amines/amides, have demonstrated that they have a higher chance of developing tumorous offspring [69]. This effect is decreased when vitamin C inhibits the nitrosation reaction required to produce carcinogens [70]. This theory can be applied to other pediatric malignancies, such as lymphoma and leukemia [71]. Regarding potential causes, vitamin C acts as an antioxidant and may shield DNA from oxidative damage, hence preventing the start of carcinogenesis. Furthermore, vitamin C has the potential to deactivate reactive metabolites inside the duodenum or stomach and inhibit the synthesis of carcinogenic N-nitroso compounds [72]. Additionally, case–control research conducted from 1995 to 2002 identified the foods consumed in the first two years of life linked to an increased risk of pediatric leukemia [73]. Nutritional information was gathered from a survey given to the child’s caretaker. Frequent fruit consumption in the first two years of life was linked to a lower chance to develop leukemia, which is diagnosed between the ages of two and fourteen. A similar pattern of decreased risk was observed when the analysis was limited to leukemia diagnosed between the ages of 2 and 5 years. These findings imply that vitamin C-rich fruits or fruit juices may lower the incidence of juvenile leukemia, particularly if ingested regularly during the first two years of life [73].

Nonetheless, other investigations have not observed a correlation between the incidence of pediatric leukemia and the consumption of fruits or fruit juices. Peters et al. [74] examined 252 leukemia cases diagnosed between the ages of one and ten from the Los Angeles County Cancer Surveillance Program (1980–1987). There was no proof that fruit or fruit-flavored beverages decreased a child’s leukemia risk. The disparity in the findings can most likely be attributed to the distinct demographic that was researched and the indirect mode of data collection. However, more research has been carried out on the potential for vitamin C to alter the chemotherapeutics’ activity in both in vitro and in vivo experimental models, and the results in promyelocytic leukemia have been interesting.

The driver oncogenic fusion protein (PML/RARα) of acute promyelocytic leukemia (APL) is a key factor in initiating APL leukemogenesis. Since the advent of all-trans retinoic acid (ATRA) and then arsenic trioxide (As_2_O_3_), the treatment of APL has changed. This is especially true for standard-risk APL with an initial white blood cell count of less than 10,000/μL, where a high cure rate can now be attained [75]. Little evidence currently suggests that vitamin C may broaden the therapeutic range of As_2_O_3_ in individuals with APL [76]. A study found that vitamin C boosts the cytotoxicity of As_2_O_3_ against HL-60 cells. Since vitamin C treatment is not cytotoxic, it may be a safe and useful chemosensitizing agent when used with As_2_O_3_-based chemotherapy [77]. As for the mechanism, it is plausible that As_2_O_3_ therapy led to increased ROS generation since vitamin C enhanced As_2_O_3_-mediated cell death. Public findings support this conclusion by showing that arsenic causes the production of ROS, which is a major factor in cell death [78,79,80]. According to a recent study, vitamin C increases the breakdown of lipid peroxidation products into fatty acid metabolites such as hydroxynonenal (HNE) when transition metals are present [81]. In light of the antiproliferative effects and the capacity to induce oxidative stress, which may lead to tumor cell apoptosis, vitamin C and As_2_O_3_ may improve the clinical outcome in APL patients since As_2_O_3_ is the first treatment choice for non-responders APL cases.

Various experimental protocols are being conducted to assess the impact of vitamin C on individuals affected by acute leukemia (Table 1).

### 3.2. Vitamin C and Transplant

Vitamin C may be important for the fragile subset of individuals receiving hematopoietic stem cell transplantation (HSCT). These individuals typically consume less food, and their serum vitamin C levels are noticeably decreased [82]. Additionally, chemotherapy can dramatically lower vitamin C concentrations in cancer patients [83], including those undergoing HSCT [84,85]. Divergent data occasionally exist regarding how vitamin C administration affects this particular population. Before obtaining HSCT, the prevalence of supplement use and the relationships between certain supplements and results were investigated. Vitamin C at > or =500 mg/day was strongly correlated with death or relapse and nonrelapse mortality in individuals with acute leukemia [86]. Thus, vitamin C supplements before therapy may raise the risk in recipients of hematopoietic stem cells who have acute leukemia. Nonetheless, vitamin C supplementation may improve some aspects of immune system function. To ascertain whether vitamin C supplementation enhances NK cell reconstitution following HSCT, Urbalejo-Ceniceros and his group [87] conducted prospective clinical research employing high-dose vitamin C. This study showed that after transplantation, patients treated with high doses of vitamin C for 100 days achieved increases in NK and CD3+ cells from day 30 to 100, reducing the rate of infections and without developing severe side effects. In contrast, the time to neutrophil recovery in a separate randomized, blinded clinical trial [88] was 11.2 days and did not differ between patients receiving the supplement and those not showing any advantages of vitamin C supplementation on neutrophil recovery and hospital admission. However, the vitamin C group appeared to experience fewer cases of bacteremia. This may be due to the baseline conditions of vitamin C’s assets. Although high blood vitamin C levels do not affect intracellular levels when these are within normal ranges, these conditions may be able to prevent or correct low intracellular vitamin C levels. The peripheral blood mononuclear cells act as a vitamin C reservoir. Furthermore, a brief period of vitamin C deficiency does not influence vitamin levels. For these reasons, the mean intracellular vitamin C concentrations in this cohort of autologous SCT patients did not change. HSCT has significant side effects, such as an elevated risk of sepsis and septic shock since there are not enough leukocytes to fight the infection. Another typical adverse effect of chemotherapy is oral mucositis, which can impair oral intake due to unpleasant mucous membrane inflammation and ulceration [89]. HSCT, radiation therapy, or chemotherapy-induced oxidative stress and ROS production are believed to trigger oral mucositis by inducing an inflammatory response [90]. Few studies have evaluated the role of vitamin C, although meta-analysis suggests that vitamins with antioxidant capabilities may assist in mitigating treatment-induced oral mucositis [91]. Early research indicates that patients with more severe mucositis typically have poorer vitamin C status [92]. Additionally, a study on six patients who had received allogeneic HSCT demonstrated an improvement in vitamin C pool after vitamin C supplementation (2 g/day for eight weeks) and a marked improvement in mucous membranes, which allowed the patients to eat without pain or restriction [93]. Therefore, the antioxidant and anti-inflammatory properties of vitamin C may lessen the frequency and intensity of side effects associated with HSCT and chemotherapy. Supporting evidence for this idea is the fact that giving high-dose intravenous vitamin C to cancer patients receiving chemotherapy has been demonstrated to reduce off-target organ toxicity [94], as well as common adverse effects of therapy, including nausea, vomiting, fatigue, and pain, all of which improve patient quality of life [95].

### 3.3. Vitamin C and Chronic Myeloid Leukemia

The clonal hematopoietic stem cell disease known as chronic myeloid leukemia (CML) is characterized by oncogenic breakpoint cluster region–Abelson (BCR–ABL1) gene fusion. Philadelphia chromosome, the product of chromosomes 9 and 22 translocations, sets it apart. The fused BCR–ABL1 oncogene is caused by this chromosomal defect, which inserts the *ABL1* gene close to the breakpoint cluster region gene. This dysregulated BCR-ABL1 protein phosphorylates multiple substrate proteins, leading to a loss of regulation in the cell cycle, abnormal proliferation, a reduction in stromal adhesion, and resistance to the programmed cell death process [96]. Some experiments have shown that Vitamin C can step in at certain points and modify the growth of CML cells. In fact, the literature indicates that greater proliferation and improved survival of cancer cells are strongly correlated with high levels of inflammatory mediators, particularly cytokines like interleukin (IL)-6 and tumor necrosis factor (TNF) [97]. A study explored the possibility of vitamin C suppressing lipopolysaccharide (LPS)-induced hyperinflammatory activation of K-562 cells [98]. Both vitamin C dosages used decreased TNF and IL-6 released in response to LPS. Furthermore, both vitamin C dosages inhibited the buildup of ATP and lowered the expression of the P2X7 receptor mRNA [98]. Thus, in K-562 cells, vitamin C reduces the hyperinflammatory state brought on by LPS by blocking ATP buildup, P2X7 receptor expression, and autophagy signaling. Several studies have assessed whether vitamin C could work in concert with other compounds that can cause the death of CML cells. Because of its notable anti-tumor action, 2-methoxy estradiol (2-ME), a bioactive metabolite of 17β-estradiol, has drawn more attention [99]. SOD suppression and apoptosis induction are thought to be the mechanisms by which 2-ME suppresses tumors. SOD inhibition results in free radical-mediated mitochondrial membrane disruption which, in turn, triggers apoptosis [100]. 2-ME causes concentration-dependent CML cell death [101,102]. An in vitro study examined how 2-ME and vitamin C worked together to cause CML cells to undergo apoptosis [103]. Apart from assessing ROS, apoptosis, and mitochondrial membrane potential (MMP), the expression of miR-223 was also identified in CML cells. Lastly, for in vivo validation, xenograft nude mice models were created. 2-ME + vitamin C therapy increased apoptosis, decreased MMP, and increased ROS concentration while inhibiting CML cell survival. Furthermore, 2-ME + vitamin C therapy increased the expression of miR-223 in CML cells, a micro-RNA that downregulates FLT3 and the PI3K/AKT pathway. Finally, 2-ME + vitamin C inhibited the growth of CML xenografts in mice [103]. All these mechanisms together favor CML apoptosis.

A fascinating aspect of the relationship between vitamin C and CML is the effect exerted by the vitamin on target therapy. Tyrosine kinase inhibitors (TKIs) have changed the treatment of CML by inhibiting the *Bcr-Abl* oncogene, which is primarily responsible for the survival of these cells [104]. However, due to the instability of the *Bcr-Abl* gene [105], mutations in the kinase domain of *Bcr-Abl* do occur. The most severe mutation is T315I, which replaces the threonine residue at position 315 with an isoleucine residue. As a result, cells with the T315I mutation are extremely resistant to some TKIs [106]. Several investigations have also been conducted to assess vitamin C’s potential impact on TKI resistance development. ROS’s ability to cause DNA lesions (such as mutations) is necessary for the onset of genetic instability in cancer cells [107,108]. Moreover, most cancer cells have low levels of antioxidants [109,110], resulting in a delicate redox balance easily upset by oxidative stress. Ascorbate/menadione (asc/men) is a system that produces ROS and causes cell death in a range of cancer cell lines. This mechanism is most likely complex and involves the inhibition of glycolysis [111], the deregulation of calcium homeostasis [112], and the disruption of a crucial protein’s chaperoning function, such as Hsp90 [113]. Because these cells lack antioxidant enzymes like catalase [114] and have increased levels of ROS, the process driving ROS generation depends on ascorbate-driven menadione–redox cycling, which causes the production of H_2_O_2_. This molecule is more harmful for cancer cells than for non-cancer cells. According to research findings, asc/men is effective against all Bcr-Abl-bearing cell lines in vitro and solid tumor models, regardless of whether the cell lines contain the T315I or E255K mutations [115]. Regarding peripheral blood leukocytes obtained from healthy donors, asc/men are less cytotoxic. The author hypothesizes that cancer cells are more susceptible to asc/men than healthy cells because they lack antioxidant enzymes, particularly catalase. The oxidative cleavage of Hsp90 and the consequent loss of its chaperone function are the mechanisms responsible for the cytotoxicity of the asc/men combination. This leads to the destruction of both wild-type and mutant Bcr-Abl proteins.

Furthermore, asc/men significantly slowed the proliferation of K562 and BaF3/Bcr-Abl-T315I cells after being implanted in mice. However, when injected into mice’s blood, asc/men had no effect on the growth of BaF3/Bcr-Abl cells. The most plausible reason is that red blood cells detoxify H_2_O_2_ and shield tissues and cells from the harm it causes [116]. Moreover, as the chaperone function of Hsp90 is necessary for the stability of the Bcr-Abl protein, one may hypothesize that asc/men would destroy cells expressing either wild-type or mutant forms of Bcr-Abl by causing Hsp90 cleavage and the destruction of its client proteins [117]. Several experimental protocols are being conducted to assess the impact of vitamin C on individuals affected by CML (Table 2).

Finally, it is interesting to note that vitamin C may help develop a practical technique to assess the effectiveness of TKIs in real time for tracking CML treatment. Caspase-3 is a recognized indicator of cellular death and can be used to assess the therapeutic efficacy of medications [118,119]. Extensive research is being conducted on sensitively detecting caspase-3 activity to improve drug resistance assessment, therapeutic decision making, and treatment monitoring [120]. Presumably, caspase-3 detection may also be a useful tool for tracking the course of CML treatment and assessing the effects of medication [121].

Due to its high sensitivity and the various energy types of excitations and detection, photoelectrochemical sensing has recently generated much attention in research [122]. An innovative approach to evaluating antileukemia drugs was reported in a study [123]. It relies on a multi-signal-amplified photoelectrochemical-sensing platform to track the activity of caspase-3, a recognized indicator of cell apoptosis. A straightforward wet chemical approach produced manganese-doped CdS@ZnS core–shell nanoparticles (Mn:CdS@ZnS), yielding a consistent photocurrent signal. These nanoparticles were used to immobilize a DEVD–biotin peptide and streptavidin-labeled alkaline phosphatise (SA-ALP) in a sequential manner via amide bonding and specific interaction between biotin and streptavidin, respectively. As the ALP degraded the substrate 2-phosphor-L-ascorbic acid (vitamin CP) to vitamin C, a more effective electron donor, the photocurrent of this sensor platform improved. This sensing platform allowed for the detection of caspase-3 activity, which allowed for the assessment of nilotinib’s effectiveness in targeting K562 CML cells [123,124]. The findings show that nilotinib can successfully cause the K562 cells to undergo apoptosis. This sensing technology demonstrated sensitive, reproducible, and steady performance when investigating K562 CML cells’ apoptosis caused by nilotinib.

### 3.4. Vitamin C and Lymphoma

Based on the well-established connection between nutrition and immunological function, there has been conjecture that diet affects the risk of non-Hodgkin lymphoma (NHL). By suppressing ROS, which are produced internally and externally in response to stimuli, a high consumption of antioxidant compounds may prevent cancer development. Antioxidants also reduce oxidative damage to lipid membranes and DNA, especially in rapidly proliferating cells like immune system cells [125]. Compared to other cells, immune system cells often contain higher amounts of substances with antioxidant qualities. The risk of NHL has been linked to consuming fruits and vegetables, which are key sources of antioxidant elements, in several studies conducted to date [126,127,128,129].

In a study of the Women’s Health Initiative on 154,363 postmenopausal women, 1104 cases of NHL were detected. The study examined the relationship between the consumption of antioxidant foods and the risk of NHL and its major subtypes. Diffuse large B-cell lymphoma (DLBCL) risk was inversely correlated with total vitamin C intake [130]. However, many unanswered questions remain on how vitamin C affects normal and lymphoma cells differently. As currently understood, ascorbate causes hydrogen peroxide-dependent cytotoxicity in lymphoma cells without deleteriously affecting healthy cells.

To ascertain the underlying mechanism of this occurrence, a study isolated parental cells of Burkitt lymphoma that were sensitive to ascorbate (JLPS cells) and resistant to it (JLPR cells) [131]. Ascorbate-induced cytotoxicity and resistance were imparted onto JLPR cells due to their enhanced glucose uptake, as evidenced by elevated glucose transporters, increased activity of antioxidant enzymes, and altered cell cycle compared to JLPS cells. Five genes (*ATF5*, *CD79B*, *MHC*, *Myosin*, and *SAP18*) with differential expression levels in vitamin C-resistant cells were linked to phosphoinositide 3 kinase, cdc42, DNA methylation, and transcriptional repression, polyamine regulation, and integrin-linked kinase signaling pathways, according to transcriptome profiles and function pathway analysis [131]. These findings revealed that JLPR cells underwent coordinated modifications to survive when subjected to the cytotoxic stress of pharmacologic ascorbate therapy.

When it comes to the ways in which vitamin C might impede the growth of lymphoma cells, an impact on epigenetic processes appears to be a key factor. DNA methylation is a crucial method for regulating gene expression. One important mechanism of cancer progression has been discovered as changes in DNA methylation that either silence or activate particular genes [132,133].

High levels of intra-tumor methylation heterogeneity and methylation disruption have been identified in DLBCL, and these findings are linked to poorer patient outcomes [134]. It has been demonstrated that low-dose DNA methyltransferase (DNMT) inhibitor therapy can rewire chemoresistant cells to become more sensitive by boosting the expression of tumor suppressor genes suppressed by DNA methylation, such as SMAD1 [135]. DNA hypermethylation has been observed due to mutations in the TET enzymes in DLBCL and peripheral T-cell (PTCL) lymphomas. According to recent research using embryonal stem cells, vitamin C is a cofactor that increases TET activity and has a binding site at the catalytic domain. Vitamin C may increase TET activity in tumor cells, which could lead to DNA demethylation, favor the expression of tumor suppressor genes, and improve chemosensitivity.

A clinical cause of TET hypofunction was suggested by the inadequate plasma vitamin C levels. These findings suggest that vitamin C may alter TET function in lymphomas.

Consequently, vitamin C shortage may worsen TET function and increase resistance [136]. There is no doubt that vitamin C’s anti-lymphoma activity involves additional action methods and targets particular cell types, such as positive cells for the Epstein–Barr virus (EBV). EBV has been linked to several cancers, such as anaplastic nasopharyngeal carcinoma, post-transplant lymphoproliferative disease, Hodgkin’s disease, and B cell lymphomas in AIDS patients [137,138]. EBV latent membrane protein 1 (LMP1) is expressed in almost all illnesses. This oncogene protein stimulates the NF-κB pathway, preventing cell death and promoting B cell growth. Tumor necrosis factor receptor-associated death domain protein (TRADD) and tumor necrosis factor receptor-associated factors (TRAFs) are bound by LMP1, which activates NF-κB to support growth transformation [138]. Oxidative stress is brought on by the EBV infection of B cells, and hydrogen peroxide is essential for preserving EBV latency [139]. Vitamin C at pharmacologic doses has been demonstrated to induce cell death at concentrations below 5 mM in various cancer cell lines, including EBV-negative Burkitt lymphoma (BL) cell lines [55,140,141]. Unlike EBV-negative BL cells or EBV-transformed lymphoblastoid cells (LCLs), EBV-positive BL cells were more vulnerable to vitamin C-induced cell death [142]. While vitamin C did not cause apoptosis in any of the cells examined, it did cause ROS and cell death.

However, while vitamin C was very successful in vitro at eliminating EBV-positive BL cells and LCLs, it was unsuccessful in an animal model and counteracted the effects of bortezomib on cell death.

An in vitro study showed that malignant B-cells are susceptible to L-ASC [140]. The overexpression of thioredoxin (TXN)-dependent antioxidant enzymes in malignant B cells may inhibit the anticancer effect of pharmacological ascorbate against them [143,144]. This may be more important in vivo, as stromal cells give cancerous cells extra redox-protective assistance [145]. However, some in vivo tests have yielded moderate outcomes, which could result from a different dosage. In an experiment, pharmacological ascorbate could remain in millimolar blood concentrations in humans after two hours of intravenous infusion, unlike in mice [146]. Clinical trial results showed that intravenous ascorbate at high doses is tolerated and non-toxic, but it has no anticancer effect when used as a monotherapy [147]. On the other hand, vitamin C might operate differently in specific types of B-cell lymphoma. Five percent to seven percent of non-Hodgkin lymphomas are Mantle cell lymphoma (MCL) [148], and evidence from studies conducted in vitro revealed that arsenic trioxide (As_2_O_3_) may be useful in treating lymphomas [149]. Patients treated with a continuous oral regimen consisting of As_2_O_3_, chlorambucil, and vitamin C were those with relapsed/refractory MCL who were not eligible for high-dose chemotherapy and had received previous regimens. In relapsed/refractory MCL, this oral regimen was effective with low toxicity, producing lasting responses in about 30% of cases [150]. Other experiments, however, produced different outcomes. While the addition of vitamin C showed relatively modest effects in some trials, in others, the chemical was even found to have a protective effect against neoplasia. The response rate to research that used As_2_O_3_ and vitamin C in combination was only 6%. For this reason, the study was stopped [151], despite earlier data indicating a potential synergy between the two compounds and demonstrating the promising activity of As_2_O_3_ in lymphoid malignancies. Lastly, a few studies have provided evidence in favor of the theory that vitamin C administration could adversely affect the therapeutic response to chemotherapy. In mice with xenogeneic tumors, vitamin C administration before doxorubicin treatment significantly decreased therapeutic efficacy [152]. Vitamin C therapy resulted in a dose-dependent reduction in apoptosis in cells exposed to chemotherapy, not because of P-glycoprotein up-regulation or vitamin C retention influenced by antineoplastics. Compared to N-acetylcysteine, vitamin C demonstrated more widespread cytoprotective properties and only mild effects on intracellular ROS, indicating a different mode of action.

Certain research on the human model of hematological malignancies has shown that vitamin C prevents treatment effectiveness by maintaining mitochondrial membrane potential when given before mechanistically distinct antineoplastic drugs [152]. These findings support the idea that vitamin C supplementation during cancer treatment may negatively impact therapeutic response.

The histology of lymphoma, the chemotherapy regimen, and the amount of vitamin C administered likely all directly affect the drug’s action.

In addition to the B-cell lymphomas covered thus far, T-cell lymphomas have also been studied in relation to vitamin C supplementation. An essential component of the treatment for various kinds of lymphomas is radiation therapy, and in this context, the relationship between vitamin C and radiotherapy appears relevant. Cells are harmed both directly and indirectly by ionizing radiation. ROS produced during radiation therapy is the primary mediator of radiation-induced damage to cells and tissues [153,154,155].

An investigation looked at the radiosensitizing impact of ascorbyl stearate (Asc-s) in 4 Gy murine T cell lymphoma (EL4) cells. Asc-s and radiation therapy stopped the cells at the S/G2 m phase of the cell cycle, which inhibited cell division and dose-dependently triggered apoptosis [156]. Additionally, it reduced the frequency of cancer stem cells overall, with a much greater decrease when combined with radiation therapy. Moreover, Asc-s and radiation therapy raised ROS levels, decreased MMP, and elevated caspase-3 activity, which led to EL4 cell apoptosis. Additionally, it greatly reduced the GSH/GSSG ratio because Asc-s bound to thiols. Thiol antioxidants in EL4 cells reversed the increase in oxidative stress brought on by Asc-s and radiation therapy. Interestingly, this redox modulation led to a time-dependent, substantial rise in protein glutathionylation. Treatment with Asc-s led to p50-NF-kB, IKK, and mutant p53 being glutathioneylated, stopping cancer growth during oxidative stress. HPLC and docking experiments show that Asc-s quenches GSH, resulting in the Asc-s + GSH adduct and further altering the GSH/GSSG ratio. In syngeneic C57/BL6 male mice, the anti-tumor impact of Asc-s in combination with radiation was investigated by injecting EL4 cells. The tumor-bearing mouse received an intraperitoneal injection of Asc-s, and after 4 Gy of radiation, they developed radio sensitization, as evidenced by considerable tumor shrinkage, as measured by the tumor burden index. Results indicating that Asc-s pre-treatment improves radio sensitization in murine models of lymphoma is corroborated by survival research [156].

These findings imply that Asc-s and ionizing radiation caused EL4 cells to enter a cell cycle block and undergo apoptosis by upsetting the redox balance via non-reversible thiol complexes with Asc-s, disrupting the permeability of the mitochondrial membrane and activating caspase-3.

However, some studies have highlighted conflicting results regarding vitamin C’s effects on treating cutaneous T-cell lymphomas (CTCLs). Malignant T cells in the skin are characteristic of these rare tumors [157]. The most prevalent CTCL is mycosis fungoides (MF), while Sézary syndrome (SS) is far less common. They make up for 53% of all cutaneous lymphomas and 2–3% of all lymphomas [158]. While there are many treatments available for the management of MF/SS, few of them are effective enough to produce long-lasting effects. Perillyl alcohol (POH) and temozolomide (TMZ) are covalently conjugated to form NEO212, a chimeric compound. POH, a naturally occurring monoterpene linked to limonene, is found in citrus fruit peel, caraway, lavender oil, cherries, cranberries, and celery seeds [159]. Numerous preclinical investigations revealed strong antitumor efficacy [160]. Silva-Hirschberg et al. [161] examined the possible anticancer effects of NEO212 on MF and SS cell lines in vitro. The cyclin D1 and c-myc proteins necessary for cell division were downregulated, but endoplasmic reticulum stress and caspase activation were brought on. Nevertheless, these NEO212-induced effects were blocked when cells were pretreated with vitamin C and beta-mercaptoethanol, indicating that ROS may be a major mediator of the actions of NEO212 [161].

This work provided a demonstration of how cell types, application techniques, and doses of vitamin C used can determine different effects on tumor cells.

### 3.5. Vitamin C and Chronic Lymphocytic Leukemia

Studies on the role of vitamin C in NHL have focused particularly on Chronic Lymphatic Leukemia (CLL), a heterogeneous malignancy marked by the accumulation of mature-looking but immunologically incompetent lymphoid cells in the spleen, marrow, peripheral blood, lymph nodes, and other tissues. CLL is the second most prevalent hematological cancer, occurring at a rate of 6.4 (6.0–6.8) cases per 100,000 people per year [162]. There are little and inconsistent nutritional epidemiology data concerning the possible role of vitamin C in the pathophysiology of CLL. Higher consumption of citrus and fruit has been linked to an increased risk of CLL, according to two European cohort studies [163,164]. However, most of the findings from cohort and case–control studies [68,127,165,166,167] are ambiguous, which may be partially explained by sample size concerns and the stark variations in fruit and vegetable diets throughout nations. Regardless, CLL has been linked to polymorphisms in the vitamin C receptor, solute carrier family 23 member 2 (SLC23A2). Three variations and one haplotype of SLC23A2 were linked to CLL in a study investigating the relationship between CLL and SNPs in SLC23A2 [168]. Additionally, a study found a correlation between a lower level of vitamin C and Binet stage C. This information may indicate vitamin C’s potential prognostic value in CLL patients [169]. However, the idea that adding vitamin C could change the patient’s response to treatment seems much more pertinent.

Arsenic trioxide can cause apoptosis in several cancers and leukemias [170]. Vitamin C has been demonstrated to enhance the cytotoxic effect of As_2_O_3_e due to its assistance in redox cycling. A study was conducted to investigate the impact of As_2_O_3_, either in combination with or without vitamin C therapy, on CLL1 cells, taking into account their prior evidence of vulnerability to oxidative stress [171]. When used alone, As_2_O_3_ and vitamin C caused cytotoxicity in CLL B cells; however, vitamin C increased As_2_O_3_’s effectiveness [172]. The pretreatment of B-CLL cells with a glutathione-reducing buthionine sulfoximine or catalase-inhibiting aminotriazole boosted As_2_O_3_/vitamin C-mediated cytotoxicity, indicating that this action is dependent on increased ROS buildup. Pretreatment with thiol antioxidants, GSH, N-acetyl cysteine, or reducing agents like catalase eliminated the cytotoxicity mediated by As_2_O_3_ and vitamin C. Moreover, As_2_O_3_ and vitamin C improved Hu1D10-mediated cell death, supporting the possible combination of As_2_O_3_/vitamin C therapy with antibodies like Hu1D10, resulting in ROS buildup [172].

There is evidence that combining vitamin C with novel medications can treat the illness without the need for chemotherapy. Recently, patient outcomes have improved with the release of B-cell receptor signaling target medication, such as BTK inhibitor (ibrutinib), PI3K inhibitor (idelalisib), and BCL2 inhibitor (venetoclax) [173]. However, with time, resistance develops to these medications due to somatic mutations acquired in the genes coding for BTK, PLCG2, and BCL2, the increased expression of anti-apoptotic genes, and interactions between the microenvironment and CLL cells [173].

Darwiche et al. [174] have demonstrated the synergistic effect between targeted therapy (ibrutinib, idelalisib, and venetoclax) and vitamin C. The same work aimed to study the anti-cancer effects of vitamin C on CLL B cells. It demonstrates how vitamin C at low doses (250 μM) causes a cytotoxic effect on CLL B cells while sparing healthy B cells. The cytotoxicity caused by vitamin C appears to be linked to the production of ROS in CLL cells and in the extracellular medium, as well as the induction of caspase-dependent apoptosis [174].

The downregulation of MCL1 expression could drive this synergistic effect by combining the two therapies. MCL-1 is an anti-apoptotic protein that contributes to the resistance to ibrutinib and venetoclax. According to Trachootham et al. [175], ROS prevents the glutathionylation of MCL-1, which would reduce its production in CLL cells. Consequently, ROS produced by vitamin C could reduce MCL-1 expression, explaining the efficacy of venetoclax and vitamin C in combined therapy. This discovery could be useful in developing logical new treatment plans that combine venetoclax with oxidative stress inducers. Similarly, by deactivating NRF2, PI3K inhibition has been connected to elevated oxidative stress in CLL cells [176]. Because AKT phosphorylation makes the protein more stable, this action could combine with ROS to target MCL-1 [177]. This could account for idelalisib and vitamin C’s combined cytotoxic effects on CLL B cells.

Moreover, ibrutinib and idelalisib cause leukemic cells to be mobilized from their protective tissue microenvironment into the blood circulation [173], which leads to a loss of the protective effects and eventually makes leukemic cells more vulnerable to cell death induced by vitamin C. This effect adds to the cytotoxic effect of ibrutinib/idelalisib.

Finally, numerous studies have attempted to explain why some CLL patients are less responsive to vitamin C therapy. It is known that the antioxidant enzyme catalase shields normal cells from oxidative stress caused by vitamin C by breaking down the produced H_2_O_2_ [178,179]. Catalase expression is decreased in CLL B-cells compared to physiological B-cells, as shown by Cavallini et al. [180]. However, a minority of patients had poorly responsive CLL B-cells to vitamin C treatment, and these patients had high catalase expression levels.

Significantly, it has been demonstrated that a more aggressive course of the disease is caused by CLL B-cells that express elevated levels of catalase [180,181]. It might be intriguing to examine the impact of vitamin C and oxidative stress in populations of CLL patients with varying prognoses—mutant and non-mutant.

**Figure 2:**
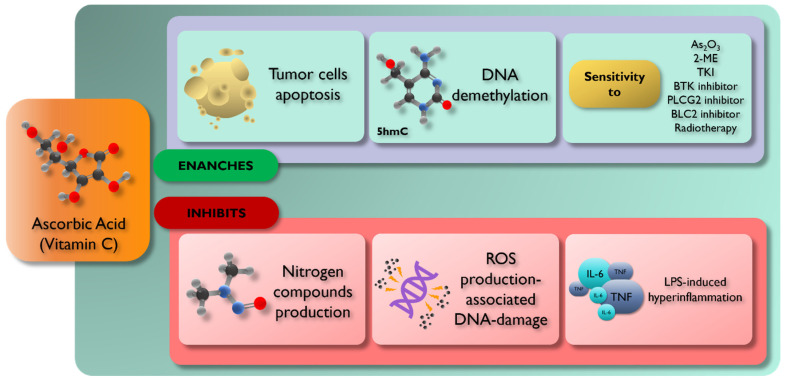
Effects of Vitamin C supplement in patients with hematological neoplastic diseases. Vitamin C plays an important role in preventing carcinogenesis by reducing ROS-associated DNA damage and nitrogen compound production. Therefore, vitamin C exerts its action on tumor cells, inducing apoptosis, DNA demethylation, and inhibiting IL-6 and TNF production. Finally, in treating various hematological tumors, the implementation of vitamin C enhances the sensitivity to chemotherapy.

Figure 2 summarizes the main effects of vitamin C on tumor cells.

### 3.6. Vitamin C and Multiple Myeloma

A stage-developed plasma cell cancer called multiple myeloma (MM) develops from smoldering MM (SMM) or monoclonal gammopathy of unknown significance (MGUS). Patient survival has significantly increased as a result of emerging therapies such as immunomodulatory medications, proteasome inhibitors, monoclonal antibodies, chimeric antigen-T/natural killer (NK) cells, bispecific T-cell engagers, selective inhibitors of nuclear export, and small-molecule targeted therapy [182]. However, because of the unavoidable drug resistance and the quick progression following a relapse, MM is still incurable.

The body’s supply of vitamin C appears to have changed in patients with MM. In one investigation, vitamin C and antioxidant enzyme levels were significantly lower in MM patients, while circulating lipid peroxides increased concurrently [183]. According to research by Xia et al. [184], when vitamin C is pharmacologically (Pvitamin C) dosed in the presence of iron, it produces a high ROS concentration that ultimately causes cell death. Patients with MGUS do not exhibit selective death from CD138+MM tumor cells obtained from MM and SMM patients. In MM xenograft mice, Pvitamin C alone or in conjunction with melphalan reduces the growth of tumors [184]. These results demonstrate the in vivo and in vitro effects of Pvitamin C on primary cancer cells and cell lines.

Moreover, it has been demonstrated that As_2_O_3_ influences the intrinsic mitochondria-associated mechanism of apoptosis in myeloma cell lines as well as the extrinsic receptor-mediated pathway [185], and this would be a compelling tool to overcome myeloma cells’ resistance to a wide range of drugs. As_2_O_3_ has been utilized in clinical trials for patients with multiple myeloma since 1998, following encouraging preclinical results [185].

Glutathione depletion caused by vitamin C may increase the cytotoxicity of As_2_O_3_ [76].

The safety, tolerability, and early efficacy of arsenic trioxide/bortezomib/vitamin C (ABC) combination therapy in patients with relapsed/refractory disease were evaluated in a multicenter, open-label, phase I/II dosage escalation research. In this extensively pretreated research group, the ABC regimen demonstrated preliminary evidence of efficacy with an objective response rate of 27%, and was well tolerated by most patients [186]. The same authors conducted a single-arm, multicenter phase 2 trial to confirm the efficacy of melphalan (BAM), vitamin C, and bortezomib for patients with recently diagnosed MM [187]. Additional research has verified these findings [188,189].

Furthermore, As_2_O_3_, in combination with vitamin C, ifosfamide, and prednisone, showed promise as a treatment for patients with many relapses and refractory to MM [190].

Lastly, it has been demonstrated that both vitamin C and bortezomib increase myeloma cells’ sensitivity to melphalan in vitro [191,192]. When doxorubicin or mitoxantrone were coupled with bortezomib, chemoresistant myeloma cell lines became 100,000 times more susceptible to melphalan’s cytotoxic and apoptosis-inducing actions and 100,000 times more sensitive to these drugs. Myeloma cells obtained from a patient who experienced a relapse following bortezomib treatment yielded comparable outcomes [193].

Some preclinical in vitro research, however, contests this assertion. Through the reduction of intracellular ROS, vitamin C was demonstrated in a recent study to shield multiple myeloma cell lines from the toxicity of arsenic trioxide [194]. Furthermore, a different study found that vitamin C reversed the in vitro cell death caused by bortezomib and suppressed the drug’s ability to block proteasome function [194]. Vitamin C’s contradictory effects have been linked to intracellular vitamin C content and the order in which these drugs are administered. Furthermore, including vitamin C in the therapy of relapsed or resistant multiple myeloma may have some bearing. When MM cells are exposed to proteasome inhibitors, they develop resistance to them, which triggers defense mechanisms such as oxidative stress reduction and the up- and down-regulation of pro- and anti-apoptotic signals. New chemicals that induce cell death to restore the response have been developed due to understanding the mechanisms by which tumor cells develop drug resistance and evade the immune system [195]. In limited research, patients with relapsed refractory multiple myeloma who were treated with carfilzomib–lenalidomide–dexamethasone and did not respond after the second cycle were given intravenous vitamin C at a pharmacologic dose of fifteen grams/week in addition to their chemotherapy regimen. For individuals who have relapsed after being resistant to chemotherapy, adding a pharmacological dosage of vitamin C to traditional chemotherapy can be a successful strategy [196]. Various experimental protocols are being conducted to assess the impact of vitamin C on individuals affected by MM (Table 3).

Lastly, using vitamin C may help manage the negative effects brought on by antimyeloma medications. BIPN, or bortezomib-induced peripheral neuropathy, appears as a debilitating side effect [197]. A study looked for cytoprotective medicines to find a way to shield nerve Schwann cells from bortezomib’s cytotoxic effects without sacrificing the drug’s anti-myeloma benefits, as rat models for BIPN have shown harm to these cells [198]. When Schwann cells are exposed to bortezomib at 30 nM or less concentrations, vitamin C almost entirely rescues the cells. Additionally, this drug can prevent morphological alterations and toxicity in cells treated with greater dosages of bortezomib [198]. The delayed addition of vitamin C following bortezomib exposure lessened the cytotoxicity in Schwann cells but not in myeloma cells. These findings imply that postponing vitamin C therapy may be crucial for BIPN prevention. Nevertheless, it should not be disregarded, as vitamin C treatment can result in notable metabolic changes. One contraindication for pharmacologic ascorbate, despite its minimal risk, is renal impairment. It is not known whether pharmaceutical ascorbate can cause kidney failure in myeloma patients who have proteinuria [199]. Renal impairment accounts for 50% of MM complications, with up to 5% requiring dialysis. Oxalate, the end product of ascorbate metabolism, is most likely the cause of clinical toxicity. Therefore, high-dose vitamin C should not be administered if creatinine clearance is less than 30 mL/min [200].

## 4. Conclusions and Perspectives

Vitamin C is a micronutrient with multiple actions and benefits. Its anti-inflammatory, antioxidant, and protective effects against DNA damage are well known today.

Vitamin C regulates both innate and adaptive immunity [4,5]. It promotes the immunosuppressive action of T-reg lymphocytes and modulates the cytokine microenvironment, also regulating the function of NK cells, eosinophils, mast cells, and basophils [6].

It also enhances the cytotoxic action of CD8+ T cells and regulates NETosis processes [23,24]. These peculiarities make vitamin C helpful in treating various autoimmune-based inflammatory diseases, such as SLE and RA. In addition to its effects on the immune system, its action in regulating ROS is fundamental and, therefore, its role as an antioxidant [29]. This peculiarity and its ability to increase DNA demethylation processes favor genomic integrity. The beneficial effects of vitamin C on epigenetic factors and the regulation of the activity of immune system cells also explain the potential role of the integration of this micronutrient for the treatment of neoplastic pathologies and, in particular, of hematological malignancies [201,202]. The antioxidant and DNA demethylation activity, as well as the inhibitory action on the production of nitrate compounds [70], could help limit damage to the genetic heritage and prevent neoplastic pathologies.

Furthermore, vitamin C inhibitory action on NF-kB [57,203,204] and the stimulation of ferroptosis mechanisms [54] are also essential in destroying tumor cells. Several studies have demonstrated immune-activating and pro-apoptotic effects. In particular, vitamin C stimulates the production and activation of immune system cells, such as NK cells, which are essential in the defense against tumor cells [205].

Moreover, there is much evidence regarding this vitamin’s ability to negatively influence the growth and survival of myeloid or lymphoid neoplastic cells.

Also interesting is the synergistic effect between vitamin C and many chemotherapeutics or target therapies used to treat hematological neoplasms. For example, supplementation increases tumor cells’ susceptibility to As_2_O_3_ [76], tyrosine kinase inhibitors [115], and BTK, PLCG2, and BLC2 inhibitors [174]. Vitamin C would also increase sensitivity to radiotherapy [156,206].

Studies regarding the therapeutic use of vitamin C molecules conjugated with nanoparticles are of great interest. Vitamin C has poor chemical stability and low bioavailability. When conjugated with lipid nanoparticles, the stability of the vitamin and cellular uptake increase. In this way, the integration is more effective at very low doses (micromoles). Likewise, higher doses (millimoles) have a cytotoxic effect, enhancing the production of H_2_O_2_ [207].

It has recently been demonstrated how polylactic glycolic acid-b-polyethylene glycol nanoparticles grafted with ascorbic acid favor the transport and increase the therapeutic activity of rivastigmine [208].

Finally, it has recently been observed that the conservation of micronutrients inside exosome-like nanovesicles improves their distribution and bioavailability [209].

This evidence could lead to synthesizing biomolecules capable of precisely conveying micronutrients, even associated with other drugs, to target cells. In this way, therapeutic effects could be obtained with doses much lower than those required for the free drug.

There is, therefore, much evidence in the literature that supports vitamin C’s effectiveness in treating both inflammatory and neoplastic pathologies, with multiple mechanisms often common. It is necessary to carry out further studies in this regard since, in the future, we could have safe and effective integrative therapy available that could have a role that is not only preventive but also adjuvant to conventional treatment. In this way, we can reduce the dose of drugs that often lead to their numerous side effects, limit the harmful effects of the latter, and improve the quality of life of these patients.

## Figures and Tables

**Table 1:** Ongoing clinical trials on the use of vitamin C in acute myeloid leukemia (www.clinicaltrials.gov, accessed on 28 February 2024).

NCT	Study Title	Intervention	Status	Study Type
NCT00329498	L-Ascorbic Acid Depletion to Treat Acute Myeloid Leukemia and Myelodysplastic Syndromes	L-Ascorbic Acid	Suspended	Interventional
NCT00184054	Trial of Arsenic Trioxide With Ascorbic Acid in the Treatment of Adult Non-Acute Promyelocytic Leukemia (APL) Acute Myelogenous Leukemia	Arsenic Trioxide; Vitamin C	Terminated with results	Interventional
NCT02877277	Epigenetics, Vitamin C and Abnormal Hematopoiesis—Pilot Study	Dietary Supplement: Vitamin C Dietary Supplement: Placebo	Completed	Interventional
NCT03999723	Combining Active and Passive DNA Hypomethylation	Dietary Supplement: vitamin C Dietary Supplement: Placebo	Recruiting	Interventional
NCT03526666	Ascorbic Acid Levels in MDS, AML, and CMML Patients	Peripheral blood collection	Completed	Observational
NCT03397173	TET2 Mutations in Myelodysplastic Syndromes and Acute Myeloid Leukemia with Azacitidine + vitamin C	Azacitidine and vitamin C	Completed	Interventional
NCT03624270	Oral Arsenic Trioxide for Newly Diagnosed Acute Promyelocytic Leukemia	Oral arsenic Trioxide, ATRA and vitamin C	Recruiting	Interventional
NCT00671697	Decitabine, Arsenic Trioxide and vitamin C for Myelodysplastic Syndromes and Acute Myeloid Leukemia	Arsenic Trioxide Decitabine	Completed	Interventional

**Table 2:** Ongoing clinical trials on the use of vitamin C in chronic myeloid leukemia (www.clinicaltrials.gov, accessed on 28 February 2024).

NCT	Study Title	Intervention	Status	Study Type
NCT00274820	Arsenic Trioxide, Ascorbic Acid, Dexamethasone, and Thalidomide in Myelofibrosis/Myeloproliferative Disorder	Vitamin C; Arsenic trioxid; dexamethasone	Completed	Interventional
NCT01670084	Nilotinib and Combination Chemotherapy in Treating Patients with Newly Diagnosed Philadelphia Chromosome-Positive Acute Lymphoblastic Leukemia or Blastic Phase Chronic Myelogenous Leukemia	Nilotinib; rituximab; cyclophosphamide	Withdrawn	Interventional
NCT00003619	Combination Chemotherapy Followed by Peripheral Stem Cell Transplantation or Isotretinoin in Treating Patients with Acute Myeloid Leukemia, Myelodysplastic Syndrome, or Acute Lymphocytic Leukemia	Filgrastim; dietary Supplement: vitamin E; busulfan	Completed	Interventional
NCT00005641	Removal of T Cells to Prevent Graft-Versus-Host Disease in Patients Undergoing Bone Marrow Transplantation	Anti-thymocyte globulin; busulfan; cyclophosphamide	Terminated	Interventional

**Table 3:** Ongoing clinical trials on the use of ascorbic acid in multiple myeloma (www.clinicaltrials.gov, accessed on 27 February 2024).

NCT	Study Title	Intervention	Status	Study Type
NCT03602235	High Dose Ascorbic Acid for Plasma Cell Disorders	Ascorbate: Melphalan	Recruiting	Interventional
NCT03964688	Effect of Vitamin C in Autologous Stem Cell Transplantations	Vitamin C; Placebos	Completed	Interventional
NCT00590603	Trisenox, Ascorbic Acid and Bortezomib in Patients with Relapsed/Refractory Multiple Myeloma	Arsenic Trioxide, vitamin C and Bortezomib	Terminated	Interventional
NCT00661544	Arsenic Trioxide with Ascorbic Acid and Melphalan for Myeloma Patients	Arsenic Trioxide; Melphalan; vitamin C	Terminated with results	Interventional
NCT00227682	Arsenic Trioxide, Thalidomide, Dexamethasone, and Ascorbic Acid in Treating Patients with Relapsed or Refractory Multiple Myeloma	Dietary Supplement: vitamin C; arsenic trioxid; dexamethasone	Terminated	Interventional
NCT00317811	Bortezomib, Ascorbic Acid, and Melphalan in Treating Patients with Newly Diagnosed Multiple Myeloma	Vitamin C; bortezomib; melphalan	Completed	Interventional
NCT00258245	Arsenic Trioxide and vitamin C combined with Bortezomib, Thalidomide, and Dexamethasone in Treating Patients with Relapsed or Refractory Multiple Myeloma or Plasma Cell Leukemia	Vitamin C; arsenic trioxid; bortezomib	Completed	Interventional
NCT00469209	Velcade, Trisenox, Vitamin C and Melphalan for Myeloma Patients	Arsenic Trioxide; Bortezomib; Melphalan	Completed with results	Interventional
NCT00112879	Arsenic Trioxide, vitamin C, Dexamethasone, and Thalidomide in Treating Patients with Multiple Myeloma	arsenic trioxide; vitamin C; dexamethasone	Withdrawn	Interventional
NCT00085345	Melphalan, Arsenic Trioxide, and Ascorbic Acid in Treating Patients with Relapsed or Refractory Multiple Myeloma	Arsenic trioxide; vitamin C; melphalan	Withdrawn	Interventional
NCT00006021	Arsenic Trioxide Plus Vitamin C in Treating Patients with Recurrent or Refractory Multiple Myeloma	Vitamin C; arsenic trioxide	Completed	Interventional
NCT01125449	Study of High Dose Intravenous (IV) Ascorbic Acid in Measurable Solid Tumor Disease	Vitamin C	Suspended	Interventional
NCT02206425	Ixazomib as a Replacement for Carfilzomib and Bortezomib for |Multiple Myeloma Patients	Melphalan; Prednison; Cyclophosphamide	Completed	Interventional
NCT02931942	Changing Over Time of Ascorbic Acid After Chemotherapy	Blood withdrawn	Active not recruiting	Observational
NCT03276481	Prospective Evaluation of Taste Function in Multiple Myeloma Patients Undergoing Autologous Hematopoietic Cell Transplantation	Diagnostic Tests: Oral microbiota assessment; Comprehensive chemical gustometry; Measurement of salivary flow	Completed	Observational
